# HEALTH-RELATED QOL IS ASSOCIATED WITH COMPLETION OF PULMONARY REHABILITATION IN OLDER VETERANS WITH COPD

**DOI:** 10.1093/geroni/igz038.1606

**Published:** 2019-11-08

**Authors:** Patricia M Bamonti, Marilyn L Moy, Christina Goodwin, Kdee Elsen, Amy Silberbogen

**Affiliations:** 1 VA Boston Healthcare System, Boston, Massachusetts, United States; 2 VA Boston Healthcare System Harvard Medical School; Boston, Massachusetts, United States; 3 Cooper University Hospital, camden, Massachusetts, United States

## Abstract

Pulmonary rehabilitation [PR] is the standard of care for Veterans with COPD. Psychosocial factors may play a role in PR participation. This study examined psychosocial factors among 253 Veterans with COPD (M age = 70.08, SD = 8.14) who completed (n = 110), did not complete (n = 75; “drop outs”), or never started (n = 68) PR. Measures completed at baseline were health-related quality of life (HRQL; Chronic Respiratory Questionnaire), depression (Beck Depression Inventory II), and exercise self-efficacy (Exercise Self-Regulatory Efficacy). One-way ANOVAs produced no significant differences in self-efficacy or depression. Significant differences were observed for HRQL, F(2,170)=10.58, p<0.001. Post hoc analysis revealed significant differences between never starters (M=69.72, SD=16.65) and completers (M=80.22, SD=19.29), p = 0.007 and differences between drop outs (M=66.69, SD=15.24) compared to completers, p < 0.001, such that better HQRL predicted completion. These findings have important clinical implications for engaging Veterans with COPD in PR.

